# Altered Basal Ganglia Network Integration in Schizophrenia

**DOI:** 10.3389/fnhum.2015.00561

**Published:** 2015-10-12

**Authors:** Mingjun Duan, Xi Chen, Hui He, Yuchao Jiang, Sisi Jiang, Qiankun Xie, Yongxiu Lai, Cheng Luo, Dezhong Yao

**Affiliations:** ^1^Key Laboratory for NeuroInformation of Ministry of Education, Center for Information in Medicine, High-Field Magnetic Resonance Brain Imaging Key Laboratory of Sichuan Province, School of Life Science and Technology, University of Electronic Science and Technology of China, Chengdu, China; ^2^The Fourth People’s Hospital of Chengdu, Chengdu, China

**Keywords:** basal ganglia network, resting-state fMRI, independent component analysis, functional connectivity, schizophrenia

## Abstract

The basal ganglia involve in a range of functions that are disturbed in schizophrenia patients. This study decomposed the resting-state data of 28 schizophrenia patients and 31 healthy controls with spatial independent component analysis and identified increased functional integration in the bilateral caudate nucleus in schizophrenia patients. Further, the caudate nucleus in patients showed altered functional connection with the prefrontal area and cerebellum. These results identified the importance of basal ganglia in schizophrenia patients. *Clinical Trial Registration:* Chinese Clinical Trial Registry. Registration number ChiCTR-RCS-14004878.

## Introduction

The basal ganglia primarily consist of four nuclei: the striatum, the globus pallidus, the subthalamic nucleus, and the substantia nigra (Parent, 1990; Luo et al., 2012). The striatum, which is divided into the caudate, the putamen, and the nucleus accumbens, is the principal anatomical component of the basal ganglia (Ballmaier et al., 2008). The basal ganglia receive afferent inputs from the different cortex areas and send projections back to the cortex via the thalamus. This neuronal loop serves as the basis of various functions of the basal ganglia, including motor, cognitive control, motivational, and emotional processing (Cropley et al., 2006). Schizophrenia is a syndrome that presents with hallucination, delusion and self-disorder late in early adulthood (Taylor, 2011; Chen et al., 2015). Many symptoms in basal ganglia-related disorders (e.g., Huntington’s disease and Parkinson’s disease), such as cognitive defects, involuntary movements, affective disturbances, and catatonia, are phenotypically similar to the symptoms observed in schizophrenia patients (Zampieri et al., 2014). This phenomenon implies the possibility of basal ganglia pathology in schizophrenia.

Structural MRI studies found volume alteration of basal ganglia in schizophrenia patients (Glenthoj et al., 2007; Mamah et al., 2007), even in never-medicated schizophrenia patients (Shihabuddin et al., 1998). Functional MRI discovered basal ganglia dysfunction in schizophrenia patients during varies task states and resting state (Walther et al., 2011; Sorg et al., 2013; Schlagenhauf et al., 2014). Other functional imaging, such as PET (Plailly et al., 2006) and SPECT (Heinz et al., 1998), also identified striatal dysfunction in schizophrenia patients.

Previous data suggest that the basal ganglia may contribute to understanding the pathophysiology of schizophrenia. The current study aimed to investigate whether and how spontaneous neuronal activity in the basal ganglia is changed in schizophrenia patients. We collected resting-state functional MRI data of patients with schizophrenia and healthy controls. The independent component analysis (ICA) is a data driven, multivariate method that can decompose the BOLD signal into different coherent resting networks, and separate the useful signal from head motion or physiological confound signals (Luo et al., 2011a, 2014; Li et al., 2015). We chose ICA to decompose the data and selected the basal ganglia network for further analysis (Luo et al., 2012). Since the basal ganglia volume may associate with treatment response to antipsychotic medication (Hutcheson et al., 2014), we collected the medication dosage information of the patients and treated it as covariation in the calculation. We hypothesized that the intrinsic basal ganglia activity changed in schizophrenia patients and these changes would relate to some symptom dimensions of schizophrenia.

## Materials and Methods

### Subjects

Twenty-nine schizophrenia patients who were diagnosed using the structured clinical interview for DSM-IV Axis I disorders – clinical version (SCID-I-CV) and 31 controls were included in this study. All patients were chronic schizophrenia patients and interviewed using the Positive and Negative Symptom Scale (PANSS). All patients received treatment with general stable doses of antipsychiotic medication. The age, gender, and education characteristics were matched between the two groups (Table 1).The exclusion criteria included a history of neurological illness, traumatic brain injury, or substance-related disorders. In addition, subjects were excluded from analysis if their scans showed extreme motion (linear shift > 2 mm, rotation >1°). The study was approved by the Ethics Committee of the Chengdu Mental Health Center in accordance with the Helsinki Declaration. Written informed consent was obtained from each subject before the study.

**Table 1:** **Demographic and clinical characteristics of the two groups**.

Characteristic	Healthy control (31)		Schizophrenia (28)		Significance	
	*M*	SD	*M*	SD	*T* value/chi-square^a^	*p*-Value (two-tailed)
Age (years)	35.19	12.68	36.54	11.46	−0.43^b^	0.67
Gender (% male)	55%		64%		0.54^c^	0.6
Education (years)	12.81	3.77	11.39	3.08	1.57^b^	0.12
Handedness (% right)	100		93			
Duration of illness (years)			12.00	8.94		
Medication dosage in CPZ equivalents (mg)			329.72	155.85		
Duration of medication (years)			12	9.33		
PANSS-P			16.54	5.77		
PANSS-N			19.61	4.13		
PANSS-G			27.71	4.34		
PANSS-total score			63.86	9.53		

**M*, mean value; SD, standard deviation; CPZ, chlorpromazine; PANSS, Positive and Negative Symptom Scale*.

*^a^Two-tailed *t*-test ^b^and chi-square tests ^c^were conducted to assess group differences for continuous and discrete variables, respectively*.

### Image Acquisition

Experiments were performed on a 3-T MRI scanner (GE DISCOVERY MR 750, USA) in Center for Information in Medicine (CIM) of University of Electronic Science and Technology of China (UESTC). The participants’ heads were immobilized with foam padding. The resting-state functional images were acquired using a standard EPI pulse sequence. The scan parameters were as follows: TR = 2000 ms, TE = 30 ms, FA = 90°, matrix size = 64 × 64, field of view = 24 cm × 24 cm, 35 slices, and slice thickness = 4 mm (no gap). A total of 255 volumes were acquired. Participants were instructed to keep their eyes closed and not fall asleep.

### Data Preprocessing

The data sets were preprocessed using SPM8 software^1^. The first five volumes were discarded for the magnetization equilibrium. Then, slice timing and head motion correction of the functional scans were performed for all the data. One patient was excluded because the translation and rotation of the head motion exceeded 2 mm and 1°. In addition, frame-wise displacement (Power et al., 2012) from every time point for each subject was calculated, and there was no difference between groups (two-sample *t*-test, *T* = 0.949, *p* = 0.347). Next, the functional data were spatially normalized (3 mm × 3 mm × 3 mm) to the EPI template. Finally, images were smoothed by an 8-mm full width at half maximum Gaussian.

### Independent Component Analysis and Component Identification

We used the GIFT software^2^ (version 1.3e) to perform a spatial ICA (Calhoun et al., 2001). To determine the number of independent components, the dimensions of the datasets from the 59 subjects were estimated using the minimum description length criterion (Li et al., 2007). Finally, 40 independent components were determined. The functional MRI data of all participants were concatenated. Then, the principal component analysis was used to reduce the temporal dimension of the aggregate dataset. The Informax algorithm was used on the reduced data to decompose them by independent component estimation. This operation was repeated 20 times in ICASSO to achieve reliable decomposition. Individual participant components were back reconstructed into single-subject space using dual-regression. Finally, the intensity values in each map were scaled to *Z* scores.

We chose components by inspecting the aggregate spatial maps and average power spectra. Three expert viewers chose the components based on the principles that the components should exhibit primary activations in the grey matter, and their time courses should be dominated by low frequency fluctuations; in addition, the components should have low spatial overlap with known vascular, ventricular, motion, and other susceptibility artefacts.

### Second-Level Analysis of the Basal Ganglia Network

To quantitatively compare the resting-state networks between the patients and controls, two-sample *t*-test was conducted for the basal ganglia network (cluster level FDR corrected, *p* < 0.05, the initial height threshold is *p* < 0.001) within the masks that resulted from the union set of the one-sample *t*-test of the two groups (*p* < 0.05).

### Functional Connectivity Analysis

We took the brain regions that exhibited significant different activation between groups as the seeds to calculate the functional connectivity map. The mean BOLD time series was extracted from the voxel which illustrated a peak value of different activation between groups and the adjacent 26 voxels. For the functional connectivity analysis, the preprocessed images were regressed using head parameters, global mean signal, white matter, cerebrospinal fluid, and linear drift signal, and the images were then band-pass filtered (0.01–0.08 Hz) (Fox et al., 2005; Luo et al., 2015). The Pearson correlation coefficients between time courses of the seeds and of each voxel in the whole brain were obtained (Luo et al., 2011b; Cao et al., 2014). The resulting coefficients were Fisher transformed to obtain *Z* scores. Voxel-wise two-sample *t*-tests were performed to obtain group differences of the functional connectivity of the seeds between two groups (cluster level FDR corrected, *p* < 0.05, the initial height threshold is *p* < 0.001). This process was performed within the masks that resulted from the union of the functional connectivity maps (one-sample *t*-test, *p* < 0.05).

### Correlations Between Functional Properties and Clinical Variables

After eliminating the singular values, partial correlations were computed between the clinical features (duration of disease and PANSS positive, negative, general psychopathology subscales, and total scores) and the *Z* value indexes of functional properties, controlling for gender, medication dosage, and education level effects. We did not regress the effect of age because of its close relationship with disease duration (*r* = 0.788, *p* < 0.05).

## Results

### Spatial Pattern of Networks in Each Group

**Figure 1:**
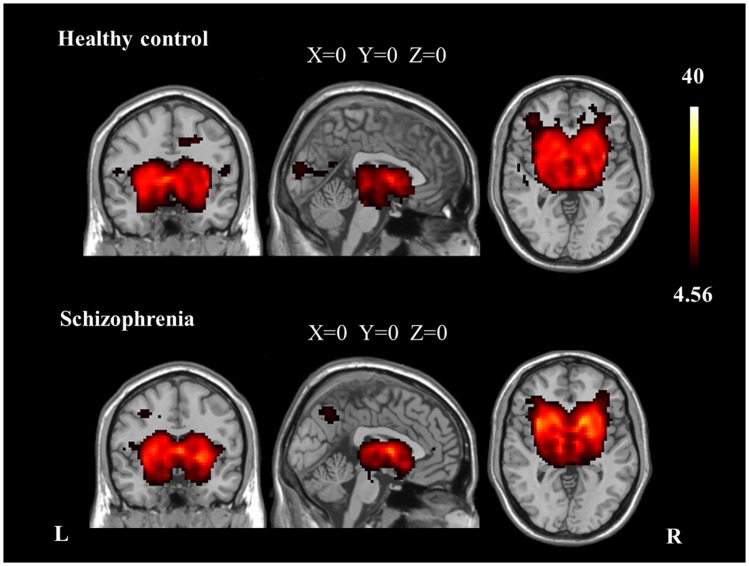
**The spatial distribution of the basal ganglia network in each group (*p* < 0.0001, uncorrected)**.

We identified the *Z* maps of the basal ganglia network according to our previous study (Luo et al., 2012). Selected components were spatially matched to previous results (Allen et al., 2011). According to the one-sample *t*-tests (*p* < 0.0001, uncorrected), the basal ganglia network primarily contained the bilateral caudate nuclei, putamen, and pallidus (Figure 1; Table 2).

**Table 2:** **Brain regions with peak value of the basal ganglia network**.

	Center (MNI)			Peak *T* value	Brain regions	BA
	*x*	*y*	*z*	
SZ	12	9	−6	30.4	Right caudate nucleus	25
	−30	9	−3	26.63	Left putamen nucleus	48
HC	27	6	5	25.73	Right putamen nucleus	48
	−23	7	−8	20.99	Left putamen nucleus	48

*BA, Brodmann area*.

### Aberrant Networks in Patients with Schizophrenia

**Figure 2:**
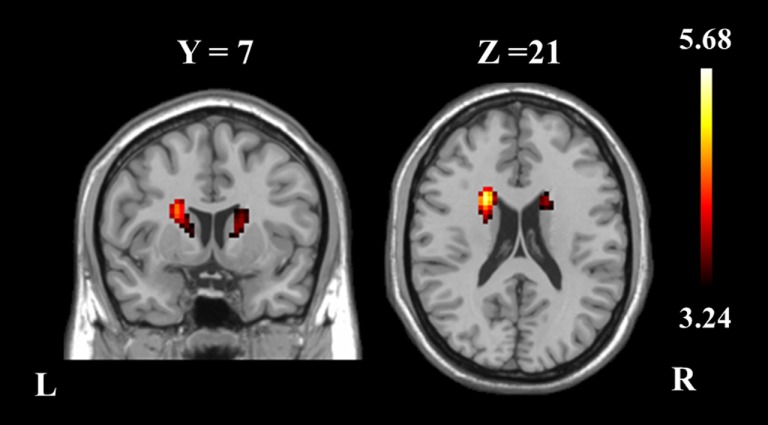
**The bilateral caudate nucleus in schizophrenia patients exhibited increased functional integration in the basal ganglia network (cluster level FDR corrected, *p* < 0.05, the initial height threshold is *p* < 0.001)**.

The two-sample *t*-test revealed differences in the basal ganglia network between the two groups. The patients with schizophrenia revealed increased functional integration of the head of the bilateral caudate nucleus (cluster level FDR corrected, *p* < 0.05, the initial height threshold is *p* < 0.001) (Figure 2; Table 3).

**Table 3:** **Brain regions with significant group differences in basal ganglia network (cluster level FDR corrected, *p* < 0.05, the initial height threshold is *p* < 0.001)**.

Brain regions	BA	Cluster size (voxels)^a^	Center (MNI)			Peak *T* value
			*x*	*y*	*z*	
Left caudate nucleus	48	141	−21	9	18	5.68
Right caudate nucleus	48	71	18	9	9	4.13

*BA, Brodmann area*.

*^a^The cluster size represents the number of voxels within the cluster*.

### Functional Connectivity Analysis

**Figure 3:**
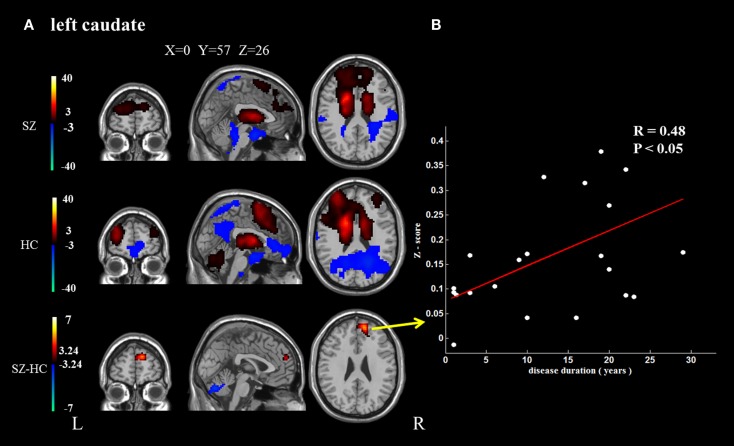
**The brain regions that exhibit significant differences between the two groups in the functional connectivity maps (cluster level FDR corrected, *p* < 0.05, the initial height threshold is *p* < 0.001)**. **(A)** The significantly altered functional connectivity between the left caudate nucleus and the superior frontal gyrus, cerebellum. Moreover, the functional connection between the left caudate nucleus and the superior frontal gyrus exhibited a positive association with the duration of the disease. **(B)** The significantly altered functional connectivity between the right caudate nucleus and the superior frontal gyrus, cerebellum and the supplementary motor area, and middle cingulate cortex. Moreover, the functional connection between the right caudate nucleus and the superior frontal gyrus exhibited a positive association with the duration of the disease.

In addition, because the caudate nucleus in the basal ganglia network revealed significant differences between the groups, we took these areas as regions of interest to build the functional connectivity map of each group. Compared with controls, the bilateral caudate nucleus exhibited enhanced functional connection with the superior frontal gyrus and decreased functional connection with the cerebellar crus in schizophrenia patients. In addition, the right caudate nucleus exhibited decreased functional connectivity with the supplementary motor area and middle cingulate cortex (cluster level FDR corrected, *p* < 0.05, the initial height threshold is *p* < 0.001) (Figure 3; Table 4).

**Table 4:** **Brain regions with significant group differences of functional connection to bilateral caudate nucleus (cluster level FDR corrected, *p* < 0.05, the initial height threshold is *p* < 0.001)**.

Seed	Brain regions	BA	Cluster size (voxels)^a^	Center (MNI)			Peak *T* value
				*x*	*y*	*z*	
Left caudate	Right medial frontal gyrus/right superior frontal gyrus	10/32	173	12	51	24	5.50
	Cerebelum_Crus1_R/cerebellum_crus2_R		729	33	−57	−39	−4.72
Right caudate	Right medial frontal gyrus/right superior frontal gyrus	10/32	375	−12	60	15	5.55
	Supplementary motor area/middle cingulum cortex	6	596	0	12	51	−5.90
	Cerebelum_Crus1_R/cerebellum_crus2_R		977	−24	−72	−54	−6.35

*BA, Brodmann area*.

*^a^The cluster size represents the number of voxels within the cluster*.

### Correlations Between Functional Properties and Clinical Variables

Positive correlations were identified between the duration of disease and the altered functional connection between the bilateral caudate nucleus and the superior frontal gyrus. No other significant correlation was found (Figure 3).

## Discussion

To test our hypothesis that aberrant basal ganglia activity might contribute to the pathophysiology of schizophrenia, resting-state functional MRI was used to study the differences of the basal ganglia network between healthy controls and schizophrenia patients. The patients exhibited increased functional integration in the bilateral caudate nucleus. Functional connectivity analysis defined enhanced functional connections between the caudate nucleus and the superior frontal gyrus, as well as decreased functional connections between the bilateral caudate nucleus and the cerebellum lobe. In addition, a decreased functional connectivity between the right caudate nucleus and the supplementary motor area and middle cingulum cortex was also defined. The results are consistent with the view that striatal dysfunction might be a fundamental element in schizophrenia patients (Howes and Kapur, 2009).

Our study found increased functional integration in the caudate nucleus in schizophrenia patients. We also identified enhanced functional connections between the caudate nucleus and the superior frontal gyrus. These results were in line with previous findings (Di Martino et al., 2008). The caudate nucleus plays a critical role in the planning and execution of behavior required for achieving complex goals (Grahn et al., 2008). A critical element of goal-directed action is expecting the outcome of the behavior. Tricomi et al. (2004) found that the head of the caudate was reliably active only under conditions in which the subjects felt the outcome depended on their response. Moreover, the caudate also participates in goal-directed behavior in social contexts. Studies have shown that the caudate is the only area to exhibit increased activation in response to benevolent reciprocity than to malevolent reciprocity (King-Casas et al., 2005). In short, the function of the caudate nucleus can be summarized as guiding behavior based on response-dependent feedback to obtain a desired outcome.

Moreover, the basal ganglia are core parts of the corticostriatal circuitry. Studies have identified three main independent loops; one of them is the association loop. This loop involves the head of the caudate and receives projections from the prefrontal lobe and presupplementary motor area (Parent, 1990). This structure had been proved by diffusion tensor fiber tracking study (Lehericy et al., 2004; Xue et al., 2014). In addition, when stimulating the prefrontal cortex, the neural activity and dopamine release in the caudate increased (Knoch et al., 2006). These studies demonstrated a clear link between the caudate and frontal area. Since the medial superior frontal cortex is also involved in decision making and cognitive control (Zhang et al., 2012), its altered functional connectivity with the caudate nucleus may implicate the impaired goal achievement behavior in schizophrenia patients, which was constantly observed in chronic schizophrenia patients. Moreover, stronger functional connectivity between the caudate and prefrontal cortex was linked to longer disease duration. This finding may reflect altered brain function aggravated by a long duration of disease.

We also identified decreased functional connectivity between the caudate nucleus and the cerebellum. Structural deficiencies within the cerebellum, especially the crus 1 and 2 parts, have been identified repeatedly in schizophrenia patients (Kuhn et al., 2012). It seems like that the cerebellum acts as a general-purpose modulator that detects pattern changes and errors in both movement and thought and provides adaptive feedback to the cerebral cortex (Andreasen and Pierson, 2008). Our finding of the reduced functional connection between the caudate nucleus and the cerebellum may help to explain the poor goal-directed performance in schizophrenia patients.

In addition, the middle cingulate cortex is a functionally heterogeneous region involved in varies cognitive and emotional processes that support goal-directed behavior (Bersani et al., 2014). The altered functional connectivity between the caudate and the middle cingulated cortex and supplementary motor area may also indicate the impaired goal-directed behavior in schizophrenia patients.

Our study has certain drawbacks. First is the relatively small sample and wide age range of the subjects in this research. In addition, the significant correlation between age and duration of disease exists in the patients group. Thus, our findings might be influenced by the effect of age. Second, this work lacks a cognitive assessment test, as the caudate nucleus, superior frontal gyrus and cerebellum all relate to cognitive functions. This weakness should be addressed in future studies.

## Author Contributions

MD, XC, DY, and CL had made a substantial contribution to the conception and design the experiment and drafting and revising the article, then they gave final approval of the version to be published; HH and QX had made a substantial contribution to the analysis and interpretation of the data, and revising the article critically, and then he gave final approval of the version to be published; YL, YJ, and SJ had made a substantial contribution to the acquisition and interpretation of the data, *t*, then they gave final approval of the version to be published.

## Conflict of Interest Statement

The authors declare that the research was conducted in the absence of any commercial or financial relationships that could be construed as a potential conflict of interest.

## Supplementary Material

The Supplementary Material for this article can be found online at http://journal.frontiersin.org/article/10.3389/fnhum.2015.00561

## Footnotes

^1^http://www.fil.ion.ucl.ac.uk/spm

^2^http://icatb.sourceforge.net/

## Abbreviations

ICA, independent component analysis; PANSS, Positive and Negative Symptom Scale.
