# Supplementary material for: Altered Basal Ganglia Network Integration in Schizophrenia
Source: Front Hum Neurosci. 2015 Oct 12;9:561. doi: 10.3389/fnhum.2015.00561 (PMC4600918; doi:10.3389/fnhum.2015.00561)
Supplement: Supplementary file 1 [file Data_Sheet_1.PDF]

Supplementary Material

Altered basal ganglia network integration in schizophrenia

Mingjun Duan[1, 2 §], Xi Chen[1 §], Hui He[1], Yuchao Jiang[1], Sisi Jiang[1], Qiankun Xie[1], Yongxiu Lai[1], Cheng Luo[1]*, Dezhong Yao[1]*

[1] Key Laboratory for NeuroInformation of Ministry of Education, Center for Information in Medicine, High-Field Magnetic Resonance Brain Imaging Key Laboratory of Sichuan Province, School of Life Science and Technology, University of Electronic Science and Technology of China, Chengdu, China
[2] Department of science and education, The Fourth People's Hospital Chengdu, Chengdu, China

[§] Contributed to this work equally

* Corresponding to Cheng Luo and Dezhong Yao, University of Electronic Science and Technology of China, Second North Jianshe Road, Chengdu, 610054, China E-mail: chengluo@uestc.edu.cn (C. Luo), dyao@uestc.edu.cn (D. Yao).

Supplementary Figures

1. The effect of the global mean signal

It is controversial whether to regress out the global mean signal as a nuisance or not. During the functional connectivity analysis, we performed the calculation both with global signal regression and without global signal regression. According to the Figure S1 and Figure S2, without global signal regression, correlation values can be relatively high across the entire brain. Thus, we would like to take the global mean signal as a nuisance covariate and regress it out.

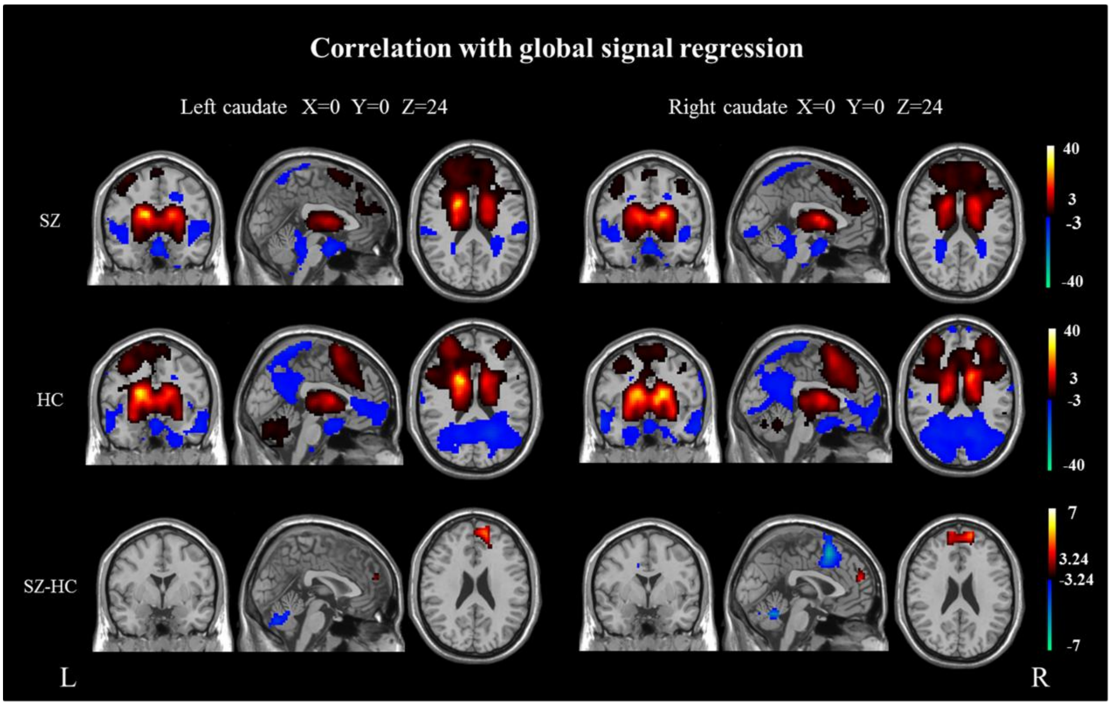

Supplementary Figure S1. The spatial distribution of the functional connectivity with global signal regression.

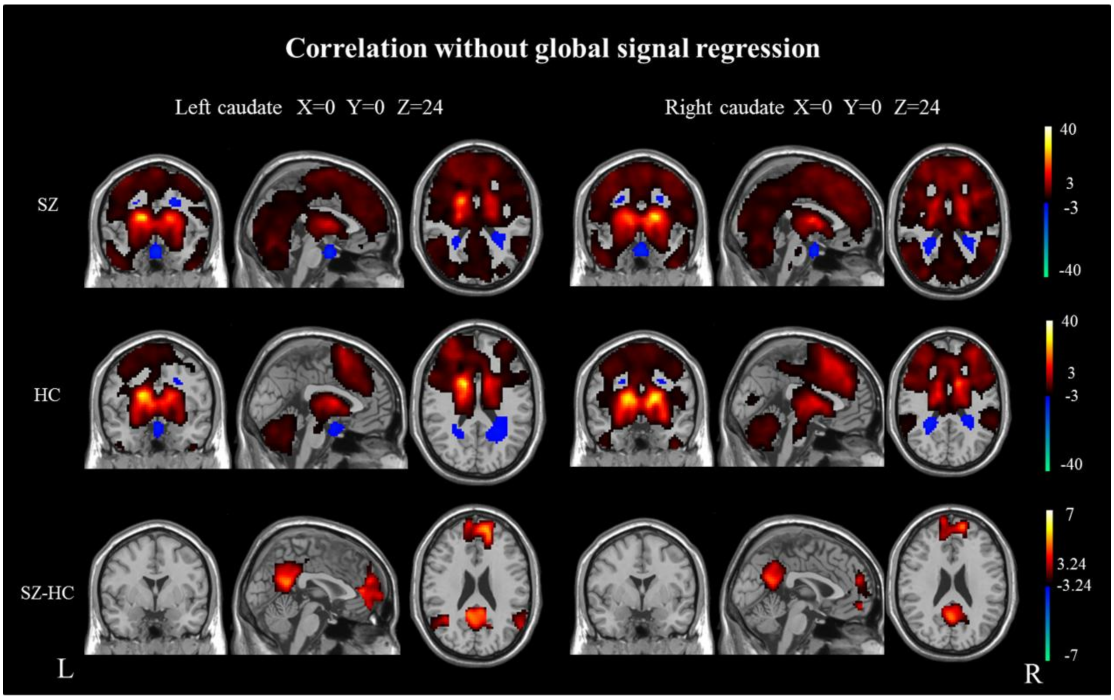

Supplementary Figure S2. The spatial distribution of the functional connectivity without global signal regression.

2. The effect of the duration of medication

In order to investigate the effect of the duration of medication, we collected this information. Correlations between functional properties and clinical variables controlling for gender, education level, medication dosage and the duration of medication were calculated (Table S1). These results were compared with the correlation coefficients that without the duration of medication regression (Table S2).

Table S1. Partial correlations between the functional properties and clinical variables, controlling for gender, education level and medication dosage

Analysis	Brain area	Duration of disease (r (p))	PANSS-P (r (p))	PANSS-N (r (p))	PANSS-G (r (p))	PANSS-total score (r (p))
ICA	Left caudate nucleus	-0.18(0.47)	0.09(0.71)	-0.42(0.07)	0.14(0.57)	-0.05(0.83)
	Right caudate nucleus	-0.21(0.39)	0.01(0.98)	-0.32(0.18)	0.35(0.14)	0.05(0.85)
Functional connectivity: left caudate nucleus	Prefrontal area	0.48(0.04)	-0.24(0.31)	0.30(0.22)	-0.13(0.58)	-0.07(0.76)
	Cerebellum	-0.39(0.10)	0.44(0.05)	-0.12(0.63)	0.31(0.19)	0.34(0.15)
Functional connectivity: right caudate nucleus	Prefrontal area	0.45(0.03)	-0.25(0.30)	0.25(0.30)	-0.11(0.65)	-0.08(0.73)
	Middle cingulum cortex	0.13(0.61)	0.23(0.35)	0.04(0.87)	-0.20(0.40)	0.03(0.89)
	Cerebellum	-0.37(0.12)	0.23(0.34)	-0.17(0.49)	0.20(0.42)	0.15(0.54)

Table S2. Partial correlations between the functional properties and clinical variables, controlling for gender, education level, medication dosage and the duration of medication

Analysis	Brain area	Duration of disease	PANSS-P	PANSS-N	PANSS-G	PANSS-total score
**ICA**	**Left caudate nucleus**	0.26(0.29)	0.02(0.95)	-0.31(0.21)	-0.04(0.86)	-0.13(0.61)
	**Right caudate nucleus**	0.07(0.78)	-0.06(0.82)	-0.22(0.37)	0.26(0.31)	0.00(0.99)
**Functional connectivity: left caudate nucleus**	**Prefrontal area**	0.60(0.01)	-0.23(0.35)	0.28(0.26)	-0.11(0.67)	-0.06(0.81)
	**Cerebellum**	-0.35(0.15)	0.43(0.07)	-0.05(0.84)	0.26(0.30)	0.32(0.19)
**Functional connectivity: right caudate nucleus**	**Prefrontal area**	0.52(0.03)	-0.23(0.36)	0.21(0.41)	-0.05(0.85)	-0.06(0.81)
	**Middle cingulum cortex**	0.14(0.58)	0.24(0.35)	0.03(0.91)	-0.20(0.42)	0.04(0.88)
	**Cerebellum**	-0.45(0.06)	0.22(0.37)	-0.15(0.55)	0.18(0.47)	0.14(0.57)

When adding the duration of medication as a covariate, the correlations between the bilateral caudate nucleus and the prefrontal area increased a little. This phenomenon may be due to the operation of regression which may reduce the individual differences. This can be seen in the Supplementary Figure S3.

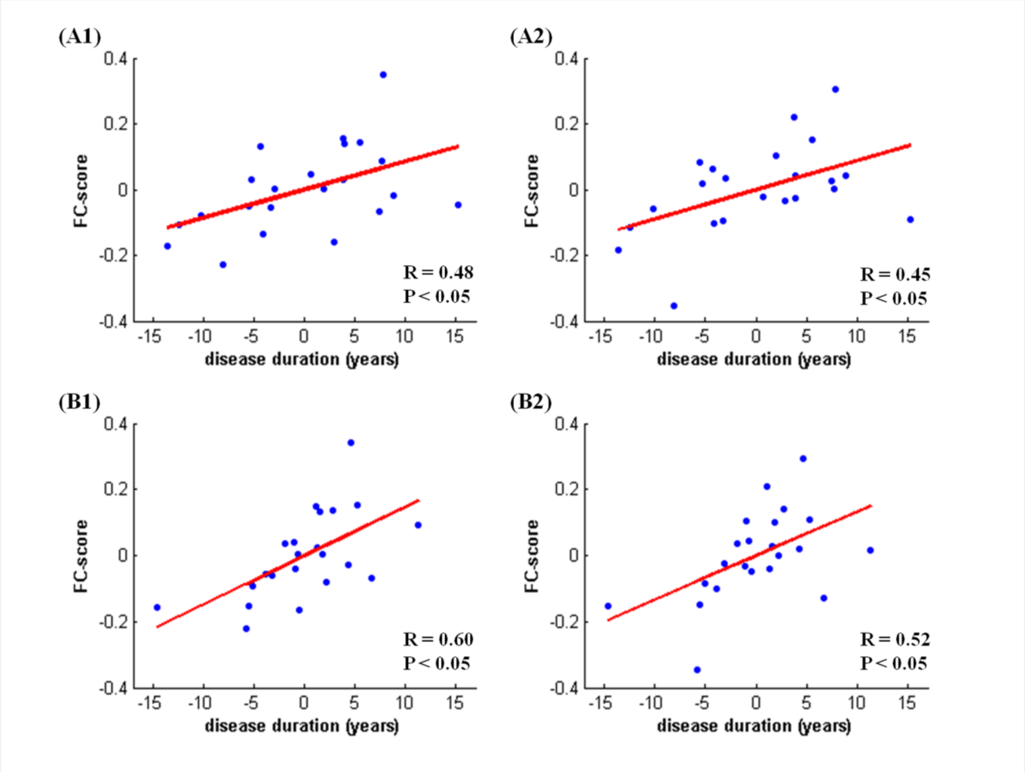

Supplementary Figure S3. The correlations between the residual of functional properties (functional properties that were regressed using controlling variables) and the residual of disease duration (disease duration that were regressed using controlling variables). (A1) the correlation between the duration of disease and the altered functional connection between the left caudate nucleus and the superior frontal gyrus, controlling for gender, education level and medication dosage. (A2) the correlation between the duration of disease and the altered functional connection between the right caudate nucleus and the superior frontal gyrus, controlling for gender, education level and medication dosage. (B1) the correlation between the duration of disease and the altered functional connection between the left caudate nucleus and the superior frontal gyrus, controlling for gender, education level, medication dosage and the duration of medication. (B2) the correlation between the duration of disease and the altered functional connection between the right caudate nucleus and the superior frontal gyrus, controlling for gender, education level, medication dosage and the duration of medication.
